# Management of postanalytical processes in the clinical laboratory according to ISO 15189:2012 Standard requirements: considerations on the review, reporting and release of results

**DOI:** 10.1515/almed-2020-0110

**Published:** 2021-01-11

**Authors:** Mᵃ Liboria López Yeste, Silvia Izquierdo Álvarez, Antonia R. Pons Mas, Luisa Álvarez Domínguez, Fernando Marqués García, Mᵃ Patrocinio Chueca Rodríguez, Aurora Blanco Font, Francisco A. Bernabeu Andreu, Ana García Álvarez, Teresa Contreras Sanfeliciano, Natalia Pascual Gómez, Lorena Sánchez Gancedo, Leonor Guiñón Muñoz

**Affiliations:** CATLAB, Barcelona, Spain; Sociedad Española de Medicina de Laboratorio (SEQCML), Comisión de Acreditación de Laboratorios, Barcelona, Spain; Servicio de Bioquímica Clínica, Hospital Universitario Miguel Servet, Zaragoza, Spain; Servicio de Análisis Clínicos, Hospital Universitari Son Espases, Mallorca, Spain; Laboratori Clínic, Hospital Universitari de Bellvitge, Barcelona, Spain; Servicio de Análisis Clínicos- Bioquímica Clínica, Hospital Universitario Puerta de Hierro, Madrid, Spain; Servicio Análisis Clínicos, Hospital Clínico San Carlos, Madrid, Spain; Servicio de Análisis Clínicos y Bioquímica Clínica, Complejo Asistencial Universitario, Salamanca, Spain; Servicio de Análisis Clínicos, Hospital Universitario de la Princesa, Madrid, Spain; Instituto de Medicina Oncológica y Molecular, Oviedo, Asturias, Spain; Hospital de la Santa Creu i Sant Pau, Barcelona, Spain; Servicio de Análisis Clínicos y Bioquímica Clínica, Laboratorio Clínico de la Metropolitana Norte, Hospital Universitario Germans Trias i Pujol, Badalona, Barcelona

**Keywords:** accreditation, clinical laboratory, ISO 15189 Standard, postanalytical process

## Abstract

The objective of this paper is to share some considerations about the management of postanalytical processes in relation to the review, reporting and release of test results in accordance with UNE-EN ISO 15189:2013 Standard requirements. The scope of this paper includes postanalytical activities and the personnel involved (laboratory management and staff). We describe the criteria and information required to review and validate analytical results and ensure that clear reports are sent to requesters. These criteria also guarantee that results are transcribed in a reliable way and that all necessary information is provided for the correct interpretation of results. Likewise, the requirements for the correct release of laboratory results are described, with special emphasis on the release of alarming or critical results. In some European countries, clinical laboratories are required to hold partial or full ISO 15189 accreditation, which is a global trend. Therefore, understanding ISO 15189 requirements is imperative for a progressive and more effective implementation of the Standard.

## Introduction

According to the UNE-EN ISO 15189:2013 Standard (hereinafter, the International Organization for Standardization [ISO] 15189), the postanalytical process can be divided into two subprocesses: a process that is completed in the laboratory, which includes the review of results, their manual recording in or online transfer to the Laboratory Information Management System (LIMS), and their release to the requesting clinician through a laboratory report [1]. The second subprocess involves activities outside the laboratory, when the requesting clinician or the treating physician receives the results, interprets them and makes a clinical decision [2].

At present, clinical laboratories are taking further action to improve postanalytical methods and communication procedures, with the aim of facilitating the interpretation of test results. Thus, laboratories increasingly provide all the information that is necessary for the correct interpretation of results, with special emphasis on the clinical significance of results [3].

Despite this, many results are improperly interpreted by the recipient, which leads to incorrect actions. According to the literature, approximately 5% of laboratory-related errors are due to a result misinterpretation, which causes 33% of delays, diagnostic errors or missed diagnoses [3]*.* Other sources indicate that the leading cause of errors in the diagnostic process correspond to errors in the interpretation of analytical results (37%), followed by errors in the test request (58%) [2], [4], [5]. However, these errors rarely compromise patient health.

It is necessary that appropriate methods are adopted for the detection, classification and prevention of postanalytical errors [6].

The objective and scope of this paper is to give recommendations for the management of postanalytical processes in accordance with the requirements of the ISO 15189 Standard, more specifically in relation to Sections 5.7, 5.8 and 5.9. In no case do these recommendations replace or extend the Standard, and they should be considered as complementary information aimed at facilitating its interpretation and application. Likewise, this paper offers recommendations to all laboratory professionals involved in postanalytical activities.

The considerations of this paper were developed by the Laboratory Accreditation Board of the Spanish Society of Laboratory Medicine (SEQC^ML^).

## Review results

The laboratory must document the procedure for the review of analytical results and ensure that it is carried out by trained, authorized personnel.

**Figure 1:**
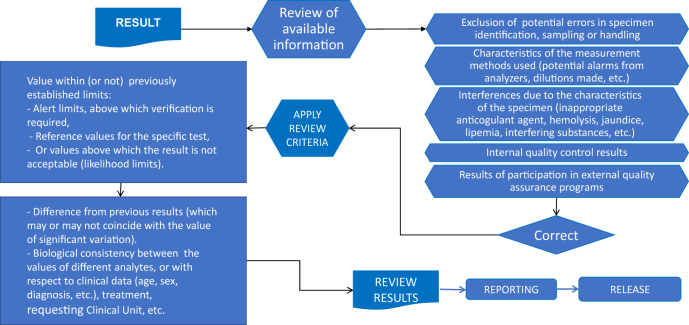
Information to be considered and criteria most used in the review of patient information.

Figure 1 details the information needed and the most common criteria for reviewing analytical results.

After the results have been reviewed in line with internal quality controls, clinical information, and previous results of the patient, results are released, together with an interpretative comment (if applicable). It is important that the predictive validity of the test is taken into account. The report may include a recommendation to perform other diagnostic tests, whether they are laboratory or not.

Occasionally, the laboratory may establish reflex/concurrent tests. These tests are only performed if a specific result is obtained and are also evaluated during the review of results.

In some laboratories, results are selected and released automatically, which is commonly referred to as “automated validation”. This requires that review criteria are well defined, documented and approved.

In emergency laboratories, partial validation of reports is commonplace, and results are separately released as they become available. This requires additional verification that new results are consistent with previous ones.

## Result reporting and edition

Once results have been reviewed, they should be sent to the requester in a clear, exact and accurate report. Laboratory professionals must ensure a reliable transcription of results (reviewed manually and automatically) and provide the necessary information for their correct interpretation. All legal requirements, as well as the recommendations and best clinical practice guidelines provided by the scientific community must also be considered.

Results can be communicated in different ways: e.g., in paper or electronic format or orally (always followed by a paper or electronic report). If results are transcribed, the process of transcription must be documented to guarantee the detection and minimization of possible errors.

The results reported by external laboratories must be identified as such in the report to ensure their traceability*.*

### Reporting

The laboratory must ensure that the report reaches an authorized person, the requesting physician or the patient (as previously arranged with the physician), while guaranteeing the confidentiality of data.

If the laboratory issues a preliminary or provisional report, it must be clearly identified as such, and the final report must include a statement that specifies that it replaces the preliminary report issued on X date.

Likewise, the laboratory must establish a procedure to inform the requester about any delay in the delivery of results, if such delay may interfere with the provision of medical care or compromise patient safety.

### Characteristics of the report

Section 5.8.2 of the Standard establishes the requirements that a report must comply with to guarantee an effective delivery of results and ensure that requesters receive all the information they need. This includes data on the specimen, the identification of alarming values, and/or the provision of interpretative comments, as detailed in Table 1.

**Table 1:** Reporting of results: characteristics and contents of reports.

Section of the report	Indication
Sample	– Type of primary sample.
	– Date of sample collection (and time, if applicable, for example, in samples for blood gas tests, or to study drug concentrations).
	– Comments on the suitability and quality of the sample in case they may potentially affect test results. To such purpose, the laboratory must define, document and communicate the criteria for accepting or rejecting a sample. These criteria will be reviewed when measurement methods are modified.
Test	– Test (and test method, where appropriate).
Patient	– Identification of the patient and location (on each page).
Requesting physician	– Identification and contact details of the requester.
Laboratory	– Identification of the laboratory where the test was performed.
	– Identification of the tests that were carried out by external laboratories.
Result	– Results and applicable units of measurement.
	– Reference intervals or clinical decision values or diagrams/nomograms.
	– Identify results within an alarming interval that may compromise patient’s safety and provide them with the treatment indicated in the text.
	– Interpretative comments on test results, where appropriate.
	– Include those interpretative comments that add value to the results obtained or are essential for their correct interpretation.
	– Other comments (tests performed in the context of a research study or development program, among others).
Reviewer	– Identification of the person who reviews and authorizes the communication of results.
Dates	– Date of request.
	– Time of sample collection, if it is relevant to the analytical determination.
	– Date and time of report.
Page	– Page number and total number of pages.
Mark	– A mark and/or note that specifies whether measurements are accredited or not.

### Report contents

The report must include the information required by the laws in force in each country. In Spain, this issue is regulated by the laws of the Health Department of each Autonomous Community and by Royal Decree 1093/2010 of September 3rd, which Annex V details the minimum set of data that should be conveyed in a test result report of the National Health System (SNS) [7].

Section 5.8.3 of the Standard indicates that reports must include information about the specimen, the patient, the test performed and the results obtained, as detailed in Table 1. Additionally, in this section, it is established that the laboratory must provide clinical guidance for the interpretation of results, including the incorporation of interpretative comments [1].

Where necessary, an interpretative comment will be included in the result report, which requires trained staff, as erroneous information or comments may compromise the conclusions drawn by the requesting physician and result in suboptimal medical care [8], [ 9]. When interpretative comments are provided by an external laboratory, the author of the comment must be duly identified.

The inclusion or not of comments will depend on the available clinical data (the clinical context of the request or patient-related factors that may influence test results such as the use of medication, for example). A comment must also be included if immediate action is necessary, if the requesting clinician needs guidance about how to interpret the results of a test, or when unexpected results are obtained. In some tests, such as genetic tests, it is necessary to include information on the limitations of the method.

The laboratory will also include interpretative comments upon physician’s request or as arranged with the requester. Therefore, the type of interpretative comment will depend on the complexity of the analytical test, the clinical Unit that requested the test and the ability of the recipient to understand interpretative comments. The lack of standardization in terms of methodology, units of measurement, reference intervals or clinical decision thresholds, added to the incorporation of new, complex laboratory tests make interpretative comments especially valuable [10].

Comments that do not add value to the interpretation of the result should be avoided (for example, stating that a value is high next to the reference interval). Furthermore, known clinical information should not be provided (for instance, adding the comment “consider hypothyroidism” in a request containing a diagnosis of hypothyroidism).

A useful comment is one that describes the normality or abnormality of a measurement result and provides and interpretation of information that contributes to follow-up. A recommendation for further testing or referral to another specialist, can be included, where appropriate [10], [11], [12].

Interpretative comments should be documented, agreed upon and standardized by the laboratory physicians responsible for reviewing results.

## Result release and delivery

**Figure 2:**
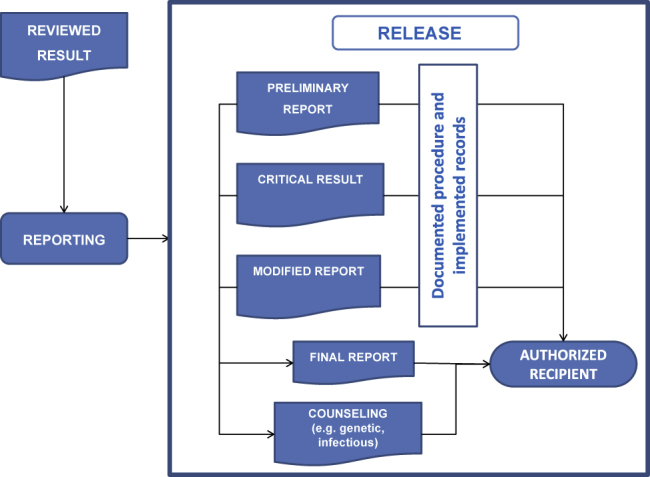
Conditions to be taken into account for the release of results.

The ISO 15189 Standard establishes that a result release protocol must be available in all laboratories. This protocol must define the professional responsible for the release of results and the person to whom results must be sent. In Section 5.9, the conditions to be taken into account in the communication process are described (see Figure 2).

The laboratory report can be either generated directly by the LIMS, or results can be sent by the LIMS to other hospital or community information systems, which will generate the report. In the event that test results are released orally, a record of results must be made to ensure their traceability (who, for whom, what, when, etc.), always followed by a written laboratory report.

The laboratory must ensure that the results of the test, the associated information and comments are incorporated to the rest of clinical information. In addition, the laboratory must also ensure that results are recorded accurately, both in electronic and paper formats, by other information systems planned to directly receive the information, while guaranteeing compliance with data protection laws (in case e-mail is used, for example). Similarly, if a new analysis or automated comment is added, it must be verified that these updates are accurately reflected in external information systems (e.g., in paper and/or electronic format).

For a limited period of time, the laboratory will inform the requesting physician of any changes made to the reference interval for a test by adding a comment in the report.

### Release of alarming results

Since Lundberg defined the concept of “critical value” in 1972 [13], the reporting of alarming results has widespread among clinical laboratories. It is the responsibility of the laboratory to establish a protocol for the correct identification and immediate communication of alarming results, which contributes to improving the care and preservation of patient safety. Moreover, reporting alarming results is mandatory in a patient-focused care setting [14].

The ISO 15189 standard requires that a documented procedure for reporting alarming results be established. The timely arrival of the report, together with confirmation of receipt, will reduce the complications resulting from a delay or failure to communicate alarming results [15]. In addition to clinician’s commitment to provide care, in accordance with the Spanish General Health Law, failure to report alarming results may constitute a severe infringement [16].

If an alarming result is detected, and before it is reported to the requester, the laboratory must verify that the test was performed in optimal analytical conditions (calibration, control results, dilutions, etc.). In addition, the laboratory must if the patient has previous critical values ​​already communicated, whether or not there are significant differences with previous results, whether diagnosis is known, or if previous results are consistent with the detected critical value. The laboratory must also assess potential errors in the identification of the sample (if the aliquot was not obtained automatically the result must be verified in the primary tube), and determine whether the sample has an acceptable quality for the results to be considered valid (presence or not of hemolysis, lypemia, etc.) [17]. The laboratory may include in the protocol specific, documented criteria for not reporting a critical result. For example, for some determinations, for some determinations, the criterion may be to have a previous result in the previous 24 h in a hospitalized patient. In this case, it is recommended to record the reason for not reporting an alarming result (availability of previous result, clinical status of the patient, if it was previously arranged with a specific Unit, etc.).

The thresholds that define a value as critical are a matter of controversy. In the “Q-Probes study 2002” [18] conducted by the College of American Pathologists (CAP), a consensus national list of critical values could not be established, but instead a generic list is provided that may serve as a starting point for each laboratory to develop its own list. The head of the laboratory is the person responsible for the design and approval of a list of threshold values, which must be agreed with the appropriate physicians. The local population they serve, the prevalence of the diseases treated, and the specialties or existing special programs must be taken into account when designing this list, and a communication procedure must be established according to the characteristics of the center [19]. A record must be kept of the critical results reported and the particularities of communication procedures (who, to whom, when and how to record data). The information to be recorded is detailed in Table 2. In addition, priority must be given to reporting the results with a greater immediate impact on the life or health of the patient, whereas the delivery of irrelevant results must be avoided. The procedure must contain provisions establishing the actions to be taken in case the test requester cannot be contacted.

**Table 2:** Details of reported alarming values.

Details of reported alarming values
– Patient ID.
– Test result.
– Type of specimen.
– Date and time of the result review.
– Date and time of the report.
– Identification of the person who releases the result.
– Identification of the person who receives the result.
– Mode of communication.
– Confirmation of receipt of the result or, in the event that communication could not be carried out within the period time established in the protocol, description of the reason.
**Recommendations**
– All reports must be documented, including failed attempts to release an alarming result.
– When applicable: record the release of alarming results in the result report.
– Where appropriate, use the LIMS and its connection with HIS to communicate critical results, as they ensure traceability, offer broad access from any site and allow prompt communication.
– Electronic release should be limited to institutional or corporative electronic mail so as to ensure reception by authorized personnel and compliance with the confidentiality of data.

Agreeing threshold values with the specialists of each Unit is especially important. For example, the same serum concentration of potassium ion substance is not interpreted in the same way by a nephrologist, an intensivist or a general practitioner, since their patients have different conditions and test results are interpreted in different clinical settings. In some studies, a consensus list of common alarming values was agreed between the laboratory and physicians of different specialties, with inconsistent conclusions about the best procedure to report these results and preferential recipients [20], [21], [22]. An order of preference of who must be sent the results must be established and documented (e.g., requesting physician, on-duty physician, nursing staff, primary care physician, 061 service, etc.) In case that a critical value cannot be released to a clinician (after the frame time for the laboratory to report a critical value has expired) and the result is directly notified to the patient or a family member, it is advisable that the laboratory recommends the patient to seek immediate medical advice and care. This procedure should always be previously agreed with clinicians and be only used in very specific cases.

Sometimes, a critical value reporting policy is established by the laboratory and the Health System. Thus, through the document “Patient Safety Strategy 2015–2020”, the Spanish National Health System (NHS) recommends “the promotion of communication between professionals through structured communication procedures that ensure the effective, timely notification of alert, alarming or critical life-threatening values” [23].

**Figure 3:**
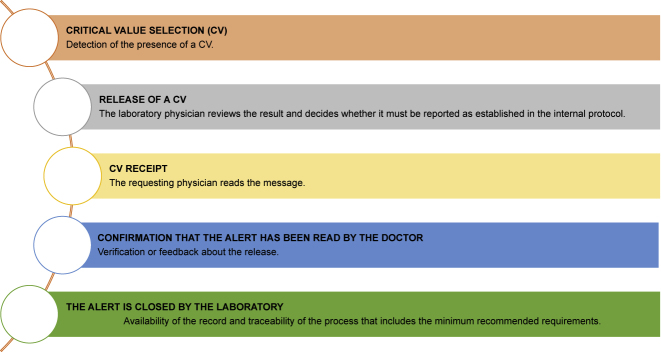
Sequence of actions for the delivery of a critical value.

In Figure 3, the sequence of actions involved in the release of critical values is detailed.

### Automated selection and reporting of results

Most analyzers perform an initial review of results automatically using the criteria previously defined (and documented) for each measure and by the laboratory physician. These criteria can be: the reference intervals (adjusted by age, sex or other conditions of the patient) or a multiple of these values; the limits that identify alarming values; the delta (change in value with respect to a previous one in a given period of time); statistically significant relationships between related tests results; diagnosis or the origin of the patient, to name a few. The review limits that statistically leave a proportion of results for manual review can also be established as criteria. The physician uses them in various ways through the computer systems that control analyzers, be it through the LIMS to which results are transmitted, or through an intermediate middleware application. In any case, the results that are automatically reviewed do not show relevant alterations or a pathology, and do not need to be reviewed by a physician.

The LIMS must allow the use of filters that identify requests from a specific Unit or containing specific values. It is recommended to use expert algorithm-based systems that identify results that need to be reviewed by the physician, since they are inconsistent or require additional comments or recommendations.

### Laboratory report corrections

The laboratory must document instructions for correcting reports. When a correction is made, a comment must be included in the report explaining the reason why the result was modified in a clear and concise way, and indicating the date of modification (time must be accessible in the laboratory) and the person responsible for such modification. Modified results and original comments must be recorded in subsequent cumulative reports (they cannot be removed from the LIMS). Modifications must be communicated to the requester, as they may have diagnostic, therapeutic or follow-up implications.

### Reporting of results from external laboratories

Section 4.5.2 of the Standard allows for the possibility of reporting results obtained by external laboratories and subcontractor consultants. According to this section, it is the responsibility of the requesting laboratory to ensure that results reach the requester.

Laboratories must have a documented procedure where external laboratories are identified and regular agreements and audits of compliance with selection and control criteria are established. A record of requests, specimens and results obtained in external laboratories must be kept to ensure the traceability of the process.

Although it should be avoided as far as possible, in those cases in which the transcription of results is necessary due to communication problems between the LIMSs of the two parties, the laboratory must ensure that results have been transcribed accurately by incorporating external laboratory reports in electronic format [e.g., in Portable Document Form (PDF) format] to electronic requests. When these reports are incorporated to the LIMS, the laboratory must adopt all measures that are necessary to guarantee unequivocal identification. Likewise, a procedure must be documented to verify compliance with the communication flow.

The standardization of external laboratory activities would facilitate quality control of activities shared by multiple centers using different LIMSs. There is limited literature available on the standardization of procedures for referring tests to external laboratories [24], [ 25]*.*

Review by a physician of the results obtained in an external laboratory is usually performed by the external laboratory, which assumes full responsibility for the results. In addition, the local laboratory must ensure that the external laboratory complies with the requirements established in the outsourcing agreement (information necessary for sample collection, storage, shipping data, the procedure for delivery of results, data to be included in reports, cost or any other relevant requirement). External laboratories are also required to use a validated measurement method, implement an internal quality control plan, and take part in intercomparison programs. The local laboratory can also review the reference values used in the external laboratory and arrange who must send results. A comment must be included in the report informing that the test was performed in the specific external center. A procedure must be established for reporting critical values where the person responsible for releasing the results is clearly identified.

When the units or values of reference are inconsistent with those of the medical history of the patient, these differences must be notified to the requesting physician in the form and format that the laboratory considers most useful for an effective release of results.

In some cases, only a part of the analytical procedure can be outsourced; the manual review and interpretation of the results of these tests can also be either outsourced or performed by the requesting laboratory. Nevertheless, it is the responsibility of the requesting laboratory to ensure that results are released appropriately.

Table 3 summarizes the requirements and records needed for the review, reporting and release of laboratory test results according to the UNE-EN ISO 15189:2013 Standard.

**Table 3:** Summary of the requirements and records needed for the review, reporting and release of laboratory test results according to the UNE-EN ISO 15189:2013 Standard.

Details to be documented	Requirements for the process	Record system (LIMS or other system)
**Review of results**	– Correct internal quality control. – Consistency with clinical information. – Consistency with previous results (specify acceptable deviation) – Criteria for re-testing. – Alerts and/or defined intervals in relation to**:** – Automated generation of results. – Physician’s manual validation. – Critical values.	– Results of calibration and control tests. – Laboratory test results. – Person responsible for the review of results. – Whether results were generated automatically or not. – Retest and original test results. – Reported critical values, reporter, date, time and recipient.
**Reporting of results**	– Electronic or paper record. – Format (see main manuscript) – Mode of release. – Expression of the result (units, comments (if applicable), reference values next to the results, among others). – Mode of reporting of delays. – Describe the way to ensure that the requester is aware of the modification.	– Changes in the way of reporting results, date and person responsible for the modification. – Changes in the expression of the result, date and person responsible for the modification. – Reporting of delays and responsible person. – Changes to report format, which includes the date, time and authorizing person.
**Release of results**	– Release flows. – Release connections. – Way to control transcripts. – Partial and final transcriptions. – Telephonic or email release process. – Special-result communication process. – Incorrect-result interruption and notification process.	– Person who communicates the results. – Contact persons, who receive the communication. – Control of transcripts. – Results communicated telephonically or by email. – Incidences/noncompliances detected due to release errors or incorrect result.
**Automated selection and reporting of results**	– Automated selection and reporting criteria.	– Validation of automated selection and reporting criteria. – Date and time of selection and reporting.
**Corrections to laboratory reports**	– Instructions for correcting an original report. – Determine how to ensure that the requester is informed about a correction.	– Modifications done including date, time and authorizing person, keeping a record of original data. – Evidence that the requester is aware that a report has been corrected.
